# Multiple Positive Solutions to Nonlinear Boundary Value Problems of a System for Fractional Differential Equations

**DOI:** 10.1155/2014/817542

**Published:** 2014-01-23

**Authors:** Chengbo Zhai, Mengru Hao

**Affiliations:** School of Mathematical Sciences, Shanxi University, Taiyuan, Shanxi 030006, China

## Abstract

By using Krasnoselskii's fixed point theorem, we study the existence of at least one or two positive solutions to a system of fractional boundary value problems given by −*D*_0^+^_^*ν*_1_^ *y*_1_(*t*) = *λ*_1_ *a*_1_(*t*)*f*(*y*_1_(*t*), *y*_2_(*t*)), − *D*_0^+^_^*ν*_2_^ *y*_2_(*t*) = *λ*_2_ *a*_2_(*t*)*g*(*y*_1_(*t*), *y*_2_(*t*)), where *D*_0^+^_^*ν*^ is the standard Riemann-Liouville fractional derivative, *ν*_1_, *ν*_2_ ∈ (*n* − 1, *n*] for *n* > 3 and *n* ∈ *N*, subject to the boundary conditions *y*_1_^(*i*)^(0) = 0 = *y*_2_^(*i*)^(0), for 0 ≤ *i* ≤ *n* − 2, and [*D*_0^+^_^*α*^ *y*_1_(*t*)]_*t*=1_ = 0 = [*D*_0^+^_^*α*^ *y*_2_(*t*)]_*t*=1_, for 1 ≤ *α* ≤ *n* − 2, or *y*_1_^(*i*)^(0) = 0 = *y*_2_^(*i*)^(0), for 0 ≤ *i* ≤ *n* − 2, and [*D*_0^+^_^*α*^ *y*_1_(*t*)]_*t*=1_ = *ϕ*_1_(*y*_1_), [*D*_0^+^_^*α*^ *y*_2_(*t*)]_*t*=1_ = *ϕ*_2_(*y*_2_), for 1 ≤ *α* ≤ *n* − 2, *ϕ*_1_, *ϕ*_2_ ∈ *C*([0,1], *R*). Our results are new and complement previously known results. As an application, we also give an example to demonstrate our result.

## 1. Introduction

The purpose of this paper is to consider the existence of multiple positive solutions for the following system of nonlinear fractional differential equations:

(1) −D0+ν1y1(t)=λ1a1(t)f(y1(t),y2(t)),−D0+ν2y2(t)=λ2a2(t)g(y1(t),y2(t)),

where *t* ∈ (0,1), *ν*_1_, *ν*_2_ ∈ (*n* − 1, *n*] for *n* > 3 and *n* ∈ *N*, and *λ*_1_, *λ*_2_ > 0, subject to a couple of boundary conditions. In particular, we first consider (1) subject to

(2) $$\begin{array}{c} y_{1}^{\left(i\right)}\left(0\right)=0=y_{2}^{\left(i\right)}\left(0\right),\;0\leq i\leq n-2,\\ {\left[D_{0^{+}}^{\alpha }y_{1}\left(t\right)\right]}_{t=1}=0={\left[D_{0^{+}}^{\alpha }y_{2}\left(t\right)\right]}_{t=1},\;1\leq \alpha \leq n-2, \end{array}$$

where *f*, *g* ∈ *C*([0, *∞*)×[0, *∞*), [0, *∞*)), and *a*_1_, *a*_2_ ∈ *C*([0,1], [0, *∞*)). We then consider the case in which the boundary conditions are changed to

(3) y1(i)(0)=0=y2(i)(0), 0≤i≤n−2,[D0+αy1(t)]t=1=ϕ1(y1),[D0+αy2(t)]t=1=ϕ2(y2),1≤α≤n−2,

where *ϕ*_1_, *ϕ*_2_ ∈ *C*([0,1], *R*).

Fractional differential equations arise in many fields, such as physics, mechanics, chemistry, economics, and engineering and biological sciences; see [1–11] for example. In recent years, the study of positive solutions for fractional differential equation boundary value problems has attracted considerable attention, and fruits from research into it emerge continuously. For a small sample of such work, we refer the reader to [12–20] and the references therein. The situation of at least one positive solution has been studied in many excellent monograph; see [12–19, 21] and other references therein. In [22], by means of Schauder fixed point theorem, Su investigated the existence of one positive solution to the following boundary value problem for a coupled system of nonlinear fractional differential equations:

(4) $$\begin{array}{c} D_{0^{+}}^{\alpha }y_{1}\left(t\right)=f\left(t,y_{2}\left(t\right),D_{0^{+}}^{\mu }y_{2}\left(t\right)\right),\;0<t<1,\\ -D_{0^{+}}^{\beta }y_{2}\left(t\right)=g\left(t,y_{1}\left(t\right),D_{0^{+}}^{\nu }y_{1}\left(t\right)\right),\;0<t<1,\\ y_{1}\left(0\right)=y_{1}\left(1\right)=y_{2}\left(0\right)=y_{2}\left(1\right)=0, \end{array}$$

where 1 < *α*, *β* < 2, *μ*, *ν* > 0, *α* − *ν* ≥ 1, *β* − *μ* ≥ 1.

In [21], Goodrich established the existence of one positive solution to problems (1)-(2) and (1), (3) by using Krasnoselskii's fixed point theorem. Different from the above works mentioned, in this paper we will present the existence of at least two positive solutions to problems (1)-(2) and (1), (3) by using the similar method presented in [21]. Moreover, under different conditions, we also present the existence of at least one positive solution to problems (1)-(2) and (1), (3) with *λ*_1_ = *λ*_2_ = 1.

## 2. Preliminaries

For the convenience of the reader, we present here some definitions, lemmas, and basic results that will be used in the proofs of our theorems.

**Definition 1 (see [23])**

Let *ν* > 0 with *ν* ∈ *R*. Suppose that *y* : [*a*, +*∞*) → *R*. Then the *ν*th Riemann-Liouville fractional integral is defined to be

(5) $$\begin{array}{c} D_{a^{+}}^{-\nu }y\left(t\right):=\frac{1}{\Gamma \left(\nu \right)}\overset{t}{\underset{a}{\int }}y\left(s\right){\left(t-s\right)}^{\nu -1}ds, \end{array}$$

whenever the right-hand side is defined. Similarly, with *ν* > 0 and *ν* ∈ *R*, we define the *ν*th Riemann-Liouville fractional derivative to be

(6) $$\begin{array}{c} D_{a^{+}}^{\nu }y\left(t\right):=\frac{1}{\Gamma \left(n-\nu \right)}\frac{d^{n}}{dt^{n}}\overset{t}{\underset{a}{\int }}\frac{y\left(s\right)}{{\left(t-s\right)}^{\nu +1-n}}ds, \end{array}$$

where *n* ∈ *N* is the unique positive integer satisfying *n* − 1 ≤ *ν* < *n* and *t* > *a*.

**Lemma 2 (see [24])**

Let *g* ∈ *C* ^*n*^([0,1]) be given. Then the unique solution to problem −*D*_0^+^_^*ν*^ *y*(*t*) = *g*(*t*) together with the boundary conditions *y* ^(*i*)^(0) = 0 = [*D*_0^+^_^*α*^ *y*(*t*)]_*t*=1_, where 1 ≤ *α* ≤ *n* − 2 and 0 ≤ *i* ≤ *n* − 2, is

(7) $$\begin{array}{c} y\left(t\right)=\overset{1}{\underset{0}{\int }}G\left(t,s\right)g\left(s\right)ds,‍ \end{array}$$

where

(8) $$\begin{array}{c} G\left(t,s\right)=\{\begin{array}{cc} \frac{t^{\nu -1}{\left(1-s\right)}^{\nu -\alpha -1}-{\left(t-s\right)}^{\nu -1}}{\Gamma \left(\nu \right)}, & 0\leq s\leq t\leq 1\\ \frac{t^{\nu -1}{\left(1-s\right)}^{\nu -\alpha -1}}{\Gamma \left(\nu \right)}, & 0\leq t\leq s\leq 1 \end{array} \end{array}$$

is the Green function for this problem.

**Lemma 3 (see [24])**

Let *G*(*t*, *s*) be as given in the statement of Lemma 2. Then one finds that

- *G*(*t*, *s*) is a continuous function on the unit square [0,1]×[0,1];
- *G*(*t*, *s*) ≥ 0 for each (*t*, *s*)∈[0,1]×[0,1];
- max⁡_*t*∈[0,1]_ *G*(*t*, *s*) = *G*(1, *s*), for each *s* ∈ [0,1].

**Lemma 4 (see [24])**

Let *G*(*t*, *s*) be as given in the statement of Lemma 2. Then there exists a constant *γ* ∈ (0,1) such that

(9) $$\begin{array}{c} \underset{t\in \left[\left(1/2\right),1\right]}{\min}G\left(t,s\right)\geq \gamma \underset{t\in \left[0,1\right]}{\max}G\left(t,s\right)=\gamma G\left(1,s\right). \end{array}$$

To prove our results, we need the following Krasnoselskii's fixed point theorem which can be seen in Guo and Lakshmikantham [25].

**Lemma 5 (see [25])**

Let *E* be a Banach space, and let *P* be a cone. Assume that *Ω*_1_,  *Ω*_2_ are open bounded subsets of *E* with 0 ∈ *Ω*_1_, $\bar{\Omega_{1}}\subset \Omega_{2}$, and let $T:P{⋂}_{\;}(\bar{\Omega_{2}}\setminus \Omega_{1})\to P$ be a completely continuous operator such that

- ||*Tu*|| ≤ ||*u*||, ∀*u* ∈ *P*⋂_ _∂*Ω*_1_, and ||*Tu*|| ≥ ||*u*||, ∀*u* ∈ P⋂_ _∂*Ω*_2_; or
- ||*Tu*|| ≥ ||*u*||, ∀*u* ∈ *P*⋂_ _∂*Ω*_1_, and ||*Tu*|| ≤ ||*u*||, ∀*u* ∈ *P*⋂_ _∂*Ω*_2_.

Then *T* has a fixed point in $P{⋂}_{\;}(\bar{\Omega_{2}}\setminus \Omega_{1})$.

## 3. Main Results

In this section, we apply Lemma 5 to study problems (1)-(2) and (1), (3), and we obtain some new results on the existence of multiple positive solutions.

### 3.1. Problem (1)-(2) in the General Case

In our considerations, let *E* represent the Banach space of *C*([0,1]) when equipped with the usual supremum norm, ||·||. Then put *X* : = *E* × *E*, where *X* is equipped with the norm ||(*y*_1_, *y*_2_)|| : = ||*y*_1_|| + ||*y*_2_|| for (*y*_1_, *y*_2_) ∈ *X*. Observe that *X* is also a Banach space (see [26]). In addition, we define two operators *T*_1_,  *T*_2_ : *X* → *E* by

(10) $$\begin{array}{c} T_{1}\left(y_{1},y_{2}\right)\left(t\right):=\lambda_{1}\overset{1}{\underset{0}{\int }}G_{1}\left(t,s\right)a_{1}\left(s\right)f\left(y_{1}\left(s\right),y_{2}\left(s\right)\right)ds,\\ T_{2}\left(y_{1},y_{2}\right)\left(t\right):=\lambda_{2}\overset{1}{\underset{0}{\int }}G_{2}\left(t,s\right)a_{2}\left(s\right)g\left(y_{1}\left(s\right),y_{2}\left(s\right)\right)ds, \end{array}$$

where *G*_1_(*t*, *s*) is the Green function of Lemma 2 with *ν* replaced by *ν*_1_ and, likewise, *G*_2_(*t*, *s*) is the Green function of Lemma 2 with *ν* replaced by *ν*_2_. Now, we define an operator *S* : *X* → *X* by

(11) S(y1,y2)(t)∶=(T1(y1,y2)(t),T2(y1,y2)(t))=(λ1∫01G1(t,s)a1(s)f(y1(s),y2(s))ds,‍  λ2∫01G2(t,s)a2(s)g(y1(s),y2(s))ds).

We claim that whenever (*y*_1_, *y*_2_) ∈ *X* is a fixed point of the operator defined in (11), it follows that *y*_1_(*t*) and *y*_2_(*t*) solve problems (1)-(2). That is, a pair of functions *y*_1_, *y*_2_ ∈ *X* is a solution of problems (1)-(2) if and only if *y*_1_, *y*_2_ is a fixed point of the operator *S* defined in (11) (see [26]).

In the following, we will look for fixed points of the operator *S*, because these fixed points coincide with solutions of problems (1)-(2). For use in the sequel, let *γ*_1_ and *γ*_2_ be the constants given by Lemma 4 associated, respectively, with the Green functions *G*_1_ and *G*_2_, and define $\tilde{\gamma }$ by $\tilde{\gamma }:=\min\{\gamma_{1},\gamma_{2}\}$, and notice that $\tilde{\gamma }\in (0,1)$.

For the sake of convenience, we set

(12) $$\begin{array}{c} f_{0}=\underset{\left(y_{1},y_{2})\to (0^{+},0^{+}\right)}{\lim}\frac{f\left(y_{1},y_{2}\right)}{y_{1}+y_{2}},\\ g_{0}=\underset{\left(y_{1},y_{2})\to (0^{+},0^{+}\right)}{\lim}\frac{g\left(y_{1},y_{2}\right)}{y_{1}+y_{2}},\\ f_{\infty }=\underset{\left(y_{1},y_{2})\to (\infty ,\infty \right)}{\lim}\frac{f\left(y_{1},y_{2}\right)}{y_{1}+y_{2}},\\ g_{\infty }=\underset{\left(y_{1},y_{2})\to (\infty ,\infty \right)}{\lim}\frac{g\left(y_{1},y_{2}\right)}{y_{1}+y_{2}}. \end{array}$$

Now we list some assumptions:

(*F*_1_) *f*_0_, *g*_0_ ∈ (0, +*∞*);
(*F*_2_) *f*_*∞*_, *g*_*∞*_ ∈ (0, +*∞*);
(*F*_3_) there are numbers Φ_1_, Φ_2_, where
  (13) Φ1≔max⁡⁡{12[∫1/21γ~G1(1,s)a1(s)f∞ds‍]−1,12[∫1/21γ~G2(1,s)a2(s)g∞ds‍]−1},Φ2≔min⁡⁡{12[∫01G1(1,s)a1(s)f0ds‍]−1,12[∫01G2(1,s)a2(s)g0ds‍]−1},

such that Φ_1_ < *λ*_1_, *λ*_2_ < Φ_2_.

Next, we define the cone *K* by

(14) K≔{(y1,y2)∈X:y1,y2≥0,min⁡t∈[(1/2),1][y1(t)+y2(t)]≥γ~||(y1,y2)||}.

**Lemma 6 (see [21])**

Let *S* be the operator defined by (11). Then *S* : *K* → *K*.

**Lemma 7**

*S* is a completely continuous operator.

**Proof**

The operator *T*_1_ : *K* → *E* is continuous in view of nonnegativeness and continuity of *G*_1_(*t*, *s*), *f*(*y*_1_, *y*_2_) and *a*_1_(*t*).

Let *Ω*⊆*K* be bounded; that is, there exists a positive constant *M* > 0 such that ||(*y*_1_, *y*_2_)|| ≤ *M*, for all (*y*_1_, *y*_2_) ∈ *Ω*. Let *L* = max⁡_0≤*t*≤1, 0≤||(*y*_1_,*y*_2_)||≤*M*_ | *a*_1_(*t*)*f*(*y*_1_(*t*), *y*_2_(*t*))|+1; then, for (*y*_1_, *y*_2_) ∈ *Ω*, we have

(15) |T1(y1,y2)(t)|  ≤λ1∫01G1(1,s)a1(s)f(y1(s),y2(s))ds‍  ≤λ1∫01G1(1,s)Lds‍  ≤LΦ2∫01G1(1,s)ds<+∞.‍

Hence, *T*_1_(*Ω*) is bounded.

On the other hand, given *ε* > 0, setting *δ* = min⁡{(1/2)(Γ(*ν*_1_)*ε*/*L*Φ_2_)^1/(*ν*_1_−1)^, *ε*Γ(*ν*_1_)/(*ν*_1_ − 1)*L*Φ_2_}, then, for each (*y*_1_, *y*_2_) ∈ *Ω*, *t*_1_, *t*_2_ ∈ [0,1], *t*_1_ < *t*_2_, and *t*_2_ − *t*_1_ < *δ*, one has |*T*_1_(*y*_1_, *y*_2_)(*t*_2_) − *T*_1_(*y*_1_, *y*_2_)(*t*_1_)|<*ε*. That is to say, *T*(*Ω*) is equicontinuity. In fact,

(16) |T1(y1,y2)(t2)−T1(y1,y2)(t1)| =|λ1∫01G1(t2,s)a1(s)f(y1(s),y2(s))ds‍  −λ1∫01G1(t1,s)a1(s)f(y1(s),y2(s))ds‍| =|λ1∫0t1[G1(t2,s)−G1(t1,s)]a1(s)f(y1(s),y2(s))ds‍  +λ1∫t1t2[G1(t2,s)−G1(t1,s)]a1(s)‍×f(y1(s),y2(s))ds  +λ1∫t21[G1(t2,s)−G1(t1,s)]a1(s)‍×f(y1(s),y2(s))ds| ≤Φ2LΓ(ν1)[∫0t1(1−s)ν1−α−1(t2ν1−1−t1ν1−1)ds‍+∫t21(1−s)ν1−α−1(t2ν1−1−t1ν1−1)ds‍+∫t1t2(1−s)ν1−α−1(t2ν1−1−t1ν1−1)ds‍] =Φ2LΓ(ν1)1ν1−α(t2ν1−1−t1ν1−1) ≤Φ2LΓ(ν1)(t2ν1−1−t1ν1−1).

In the following, we divide the proof into two cases.

*Case 1*. If *δ* ≤ *t*_1_ < *t*_2_ < 1, then we have

(17) |T1(y1,y2)(t2)−T1(y1,y2)(t1)|  ≤Φ2LΓ(ν1)(t2ν1−1−t1ν1−1)  =Φ2LΓ(ν1)(ν1−1)(t2−t1)tξν1−2  <Φ2LΓ(ν1)(ν1−1)δ≤ε,

where *t*_*ξ*_ ∈ (*t*_1_, *t*_2_).

*Case 2*. If 0 ≤ *t*_1_ < *δ*, *t*_2_ < 2*δ*, then we have

(18) |T1(y1,y2)(t2)−T1(y1,y2)(t1)|  ≤Φ2LΓ(ν1)(t2ν1−1−t1ν1−1)  ≤Φ2LΓ(ν1)t2ν1−1<Φ2LΓ(ν1)(2δ)ν1−1≤ε.

By the means of the Arzela-Ascoli theorem, we have that *T*_1_ is completely continuous. Similarly, *T*_2_ is completely continuous. Consequently, *S* : *K* → *K* is a completely continuous operator. This completes the proof.

In [21], Goodrich established the following result.

**Theorem 8 (see Theorem 3.3 in [21])**

Suppose that (*F*_1_)–(*F*_3_) are satisfied. Then problem (1)-(2) has at least one positive solution.

From Theorem 8, the following problem is natural: whether we can obtain some conclusions or not, if *f*_0_ = *f*_*∞*_ = *g*_0_ = *g*_*∞*_ = 0  or  *f*_0_ = *f*_*∞*_ = *g*_0_ = *g*_*∞*_ = *∞*? In the rest of this paper, we give some answers to this problem.

For the sake of convenience, we make some assumptions:

(*H*_1_) there exist constants *ρ*_1_, *A*_1_ > 0, such that
  (19) $$\begin{array}{c} f\left(y_{1},y_{2}\right),g\left(y_{1},y_{2}\right)<A_{1}^{-1}\rho_{1}\;\text{for}\;\;0\leq ||\left(y_{1},y_{2}\right)||\leq \rho_{1}; \end{array}$$
(*H*_2_) there exist constants *ρ*_2_, *A*_2_ > 0, such that
  (20) $$\begin{array}{c} f\left(y_{1},y_{2}\right),g\left(y_{1},y_{2}\right)\geq A_{2}^{-1}\rho_{2}\;\text{for}\;\;\tilde{\gamma }\rho_{2}\leq ||\left(y_{1},y_{2}\right)||\leq \rho_{2}; \end{array}$$
  (*P*_1_) there are numbers Λ_1_, Λ_2_, where
  (21) Λ1≔max⁡{12[∫1/21γ~G1(1,s)a1(s)ds‍]−1,12[∫1/21γ~G2(1,s)a2(s)ds‍]−1},Λ2≔min⁡{A12[∫01G1(1,s)a1(s)ds‍]−1,A12[∫01G2(1,s)a2(s)ds‍]−1},
 such that Λ_1_ < *λ*_1_,  *λ*_2_ < Λ_2_;
(*P*_2_) there are numbers Λ_3_,  Λ_4_, where
  (22) Λ3≔max⁡⁡{A22[∫1/21G1(1,s)a1(s)ds‍]−1,A22[∫1/21G2(1,s)a2(s)ds‍]−1},Λ4≔min⁡⁡{12[∫01G1(1,s)a1(s)ds‍]−1,12[∫01G2(1,s)a2(s)ds]−1},
 such that Λ_3_ < *λ*_1_,  *λ*_2_ < Λ_4_.

**Theorem 9**

Suppose that *f*_0_ = *f*_*∞*_ = *g*_0_ = *g*_*∞*_ = *∞* and (*H*_1_), (*P*_1_) are satisfied. Then problem (1)-(2) has at least two positive solutions (*y*_1_^0^, *y*_2_^0^), $\left({\bar{y}}_{1},{\bar{y}}_{2}\right)$, such that $0<||\left(y_{1}^{0},y_{2}^{0}\right)||<\rho_{1}<||\left({\bar{y}}_{1},{\bar{y}}_{2}\right)||$.

**Proof**

From Lemma 7, *S* is a completely continuous operator. At first, in view of *f*_0_ = *g*_0_ = *∞*, we have *f*(*y*_1_, *y*_2_) ≥ *M*(*y*_1_ + *y*_2_), for 0 < ||(*y*_1_, *y*_2_)|| < *r*_1_* < *ρ*_1_; *g*(*y*_1_, *y*_2_) ≥ *M*(*y*_1_ + *y*_2_), for 0 < ||(*y*_1_, *y*_2_)|| < *r*_2_* < *ρ*_1_, where *M* satisfies *M* ≥ 1. Set *ρ*_0_ : = min⁡{*r*_1_*, *r*_2_*}. So we define *Ω*_*ρ*_0__ by *Ω*_*ρ*_0__ : = {(*y*_1_, *y*_2_) ∈ *X* : ||(*y*_1_, *y*_2_)|| < *ρ*_0_}. Then for each (*y*_1_, *y*_2_) ∈ *K*⋂∂*Ω*_*ρ*_0__, we find that

(23) T1(y1,y2)(1)=λ1∫01G1(1,s)a1(s)f(y1(s),y2(s))ds‍≥λ1∫1/21G1(1,s)a1(s)f(y1(s),y2(s))ds‍≥λ1∫1/21G1(1,s)a1(s)M(y1(s)+y2(s))ds‍≥λ1∫1/21γ~G1(1,s)a1(s)M||(y1,y2)||ds‍≥M2||(y1,y2)||≥12||(y1,y2)||.

So ||*T*_1_(*y*_1_, *y*_2_)|| ≥ (1/2)||(*y*_1_, *y*_2_)|| for (*y*_1_, *y*_2_) ∈ *K*⋂∂*Ω*_*ρ*_0__.

Similarly, we find that ||*T*_2_(*y*_1_, *y*_2_)|| ≥ (1/2)||(*y*_1_, *y*_2_)|| for (*y*_1_, *y*_2_) ∈ *K*⋂∂*Ω*_*ρ*_0__. Consequently,

(24) ||S(y1,y2)||=||(T1(y1,y2),T2(y1,y2))||=||T1(y1,y2)||+||T2(y1,y2)||≥||(y1,y2)||,

whenever (*y*_1_, *y*_2_) ∈ *K*⋂∂*Ω*_*ρ*_0__. Thus, *S* is cone expansion on *K*⋂∂*Ω*_*ρ*_0__.

Next, since *f*_*∞*_ = *g*_*∞*_ = *∞*, we have *f*(*y*_1_, *y*_2_) ≥ *M*_1_(*y*_1_ + *y*_2_) for *y*_1_ + *y*_2_ ≥ *r*_1_** > *ρ*_1_;   *g*(*y*_1_, *y*_2_) ≥ *M*_1_(*y*_1_ + *y*_2_) for *y*_1_ + *y*_2_ ≥ *r*_2_** > *ρ*_1_, where *M*_1_ satisfies *M*_1_ ≥ 1. Set *ρ*_10_ : = max⁡{*r*_1_**, *r*_2_**}. Let $\rho_{0}^{*}=\max\{2\rho_{1},\left(\rho_{10}/\tilde{\gamma }\right)\}$ and *Ω*_*ρ*_0_*_ : = {(*y*_1_, *y*_2_) ∈ *X* : ||(*y*_1_, *y*_2_)|| < *ρ*_0_*}. Then (*y*_1_, *y*_2_) ∈ *K*⋂∂*Ω*_*ρ*_0_*_ implies

(25) y1(t)+y2(t)≥min⁡t∈[(1/2),1][y1(t)+y2(t)]≥γ~||(y1,y2)||=γ~ρ0∗≥ρ10.

So we obtain

(26) T1(y1,y2)(1)=λ1∫01G1(1,s)a1(s)f(y1(s),y2(s))ds‍≥λ1∫1/21G1(1,s)a1(s)f(y1(s),y2(s))ds‍≥λ1∫1/21G1(1,s)a1(s)M1(y1(s)+y2(s))ds‍≥λ1∫1/21γ~G1(1,s)a1(s)M1||(y1,y2)||ds‍≥M12||(y1,y2)||≥12||(y1,y2)||.

So ||*T*_1_(*y*_1_, *y*_2_)|| ≥ (1/2)||(*y*_1_, *y*_2_)|| for (*y*_1_, *y*_2_) ∈ *K*⋂∂*Ω*_*ρ*_0_*_.

Similarly, we find that ||*T*_2_(*y*_1_, *y*_2_)|| ≥ (1/2)||(*y*_1_, *y*_2_)|| for (*y*_1_, *y*_2_) ∈ *K*⋂∂*Ω*_*ρ*_0_*_.

Consequently, ||*S*_2_(*y*_1_, *y*_2_)|| ≥ ||(*y*_1_, *y*_2_)||, whenever (*y*_1_, *y*_2_) ∈ *K*⋂∂*Ω*_*ρ*_0_*_. Thus, S is cone expansion on *K*⋂∂*Ω*_*ρ*_0_*_.

Finally, let *Ω*_*ρ*_1__ : = {(*y*_1_, *y*_2_) ∈ *X* : ||(*y*_1_, *y*_2_)|| < *ρ*_1_}. For (*y*_1_, *y*_2_) ∈ *K*⋂∂*Ω*_*ρ*_1__, from (*H*_1_), (*P*_1_), we have

(27) ||T1(y1,y2)||=λ1∫01G1(1,s)a1(s)f(y1(s),y2(s))ds‍≤A12[∫01G1(1,s)a1(s)ds‍]−1 ×∫01G1(1,s)a1(s)dsA1−1ρ1‍=ρ12=12||(y1,y2)||.

Similarly, we find that ||*T*_2_(*y*_1_, *y*_2_)|| ≤ (1/2)||(*y*_1_, *y*_2_)|| for (*y*_1_, *y*_2_) ∈ *K*⋂∂*Ω*_*ρ*_1__.

Consequently, ||*S*(*y*_1_, *y*_2_)|| ≤ ||(*y*_1_, *y*_2_)||, whenever (*y*_1_, *y*_2_) ∈ *K*⋂∂*Ω*_*ρ*_1__. Thus, *S* is cone compression on *K*⋂∂*Ω*_*ρ*_1__.

So, from Lemma 5, *S* has a fixed point $\left(y_{1}^{0},y_{2}^{0})\in K{⋂}_{}({\bar{\Omega }}_{\rho_{1}}\setminus \Omega_{\rho_{0}}\right)$ and a fixed point $\left({\bar{y}}_{1},{\bar{y}}_{2})\in K{⋂}_{}({\bar{\Omega }}_{\rho_{0}^{*}}\setminus \Omega_{\rho_{1}}\right)$. Both are positive solutions of BVP (1)-(2) with

(28) $$\begin{array}{c} 0<||\left(y_{1}^{0},y_{2}^{0}\right)||<\rho_{1}<||\left({\bar{y}}_{1},{\bar{y}}_{2}\right)||. \end{array}$$

The proof is complete.

**Theorem 10**

Suppose that *f*_0_ = *f*_*∞*_ = *g*_0_ = *g*_*∞*_ = 0 and (*H*_2_), (*P*_2_) are satisfied. Then problem (1)-(2) has at least two positive solutions (*y*_1_^0^, *y*_2_^0^), $\left({\bar{y}}_{1},{\bar{y}}_{2}\right)$, such that $0<||\left(y_{1}^{0},y_{2}^{0}\right)||<\rho_{2}<||\left({\bar{y}}_{1},{\bar{y}}_{2}\right)||$.

**Proof**

At first, in view of *f*_0_ = *g*_0_ = 0, we have *f*(*y*_1_, *y*_2_) < *ε*(*y*_1_ + *y*_2_), *g*(*y*_1_, *y*_2_) < *ε*(*y*_1_ + *y*_2_), for 0 < ||(*y*_1_, *y*_2_)|| ≤ *ρ* < *ρ*_2_, where *ε* satisfies *ε* ≤ 1. Let *Ω*_*ρ*_ : = {(*y*_1_, *y*_2_) ∈ *X* : ||(*y*_1_, *y*_2_)|| < *ρ*}.

Then for each (*y*_1_, *y*_2_) ∈ *K*⋂∂*Ω*_*ρ*_, we find that

(29) ||T1(y1,y2)||=λ1∫01G1(1,s)a1(s)f(y1(s),y2(s))ds‍≤λ1∫01G1(1,s)a1(s)ε(y1(s)+y2(s))ds‍≤λ1∫01G1(1,s)a1(s)ε||(y1,y2)||ds‍≤ε2||(y1,y2)||≤12||(y1,y2)||.

Like Theorem 9, we get ||*S*(*y*_1_, *y*_2_)|| ≤ ||(*y*_1_, *y*_2_)|| for (*y*_1_, *y*_2_) ∈ *K*⋂∂*Ω*_*ρ*_.

Next, in view of *f*_*∞*_ = *g*_*∞*_ = 0, we have *f*(*y*_1_, *y*_2_) < *ε*_1_(*y*_1_ + *y*_2_), *g*(*y*_1_, *y*_2_) < *ε*_1_(*y*_1_ + *y*_2_), for *y*_1_ + *y*_2_ ≥ *ρ*′ > *ρ*_2_, where *ε*_1_ satisfies *ε*_1_ ≤ 1. We consider two cases.

*Case 1.* Suppose that *f* is unbounded; there exists ρ* > *ρ*′ such that

(30) f(y1,y2)≤f(y1∗,y2∗) for  0≤||(y1,y2)||≤ρ∗,||(y1∗,y2∗)||=ρ∗.

Since ρ* > *ρ*′, one has *f*(*y*_1_, *y*_2_) ≤ *f*(*y*_1_*, *y*_2_*) < *ε*_1_(*y*_1_* + *y*_2_*) for 0 ≤ ||(*y*_1_, *y*_2_)|| ≤ ρ*. Then, for (*y*_1_, *y*_2_) ∈ *K* and ||(*y*_1_, *y*_2_)|| = ρ*, we obtain

(31) ||T1(y1,y2)||=λ1∫01G1(1,s)a1(s)f(y1(s),y2(s))ds‍≤λ1∫01G1(1,s)a1(s)ε1(y1(s)+y2(s))ds‍≤λ1∫01G1(1,s)a1(s)ε1||(y1,y2)||ds‍≤ε12||(y1,y2)||≤12||(y1,y2)||.

*Case 2.* Suppose that *f* is bounded; there exists *L*_1_ such that *f*(*y*_1_, *y*_2_) ≤ *L*_1_ for all (*y*_1_, *y*_2_) ∈ *K*. Taking ρ* ≥ max⁡{2*ρ*_2_, *L*_1_}, for (*y*_1_, *y*_2_) ∈ *K* and ||(*y*_1_, *y*_2_)|| = ρ*, we obtain

(32) ||T1(y1,y2)||=λ1∫01G1(1,s)a1(s)f(y1(s),y2(s))ds‍≤λ1∫01G1(1,s)a1(s)L1ds‍≤L12≤ρ∗2=12||(y1,y2)||.

Hence, in either case, we always may set *Ω*_ρ*_ : = {(*y*_1_, *y*_2_) ∈ *X* : ||(*y*_1_, *y*_2_)|| < ρ*} such that ||*T*_1_(*y*_1_, *y*_2_)|| ≤ (1/2)||(*y*_1_, *y*_2_)|| for (*y*_1_, *y*_2_) ∈ *K*⋂∂*Ω*_ρ*_. Like Theorem 9, we get ||*S*(*y*_1_, *y*_2_)|| ≤ ||(*y*_1_, *y*_2_)||, for (*y*_1_, *y*_2_) ∈ *K*⋂∂*Ω*_ρ*_.

Finally, set *Ω*_*ρ*_2__ : = {(*y*_1_, *y*_2_) ∈ *X* : ||(*y*_1_, *y*_2_)|| < *ρ*_2_}. Then (*y*_1_, *y*_2_) ∈ *K*⋂∂*Ω*_*ρ*_2__ implies

(33) y1(t)+y2(t)≥min⁡t∈[(1/2),1][y1(t)+y2(t)]≥γ~||(y1,y2)||=γ~ρ2.

Hence we have

(34) T1(y1,y2)(1)=λ1∫01G1(1,s)a1(s)f(y1(s),y2(s))ds‍≥λ1∫1/21G1(1,s)a1(s)A2−1ρ2ds‍≥A22A2−1ρ2=ρ22=12||(y1,y2)||.

Consequently, ||*T*_1_(*y*_1_, *y*_2_)|| ≥ (1/2)||(*y*_1_, *y*_2_)|| for (*y*_1_, *y*_2_) ∈ *K*⋂∂*Ω*_*ρ*_2__. Like Theorem 9, we get ||*S*(*y*_1_, *y*_2_)|| ≥ ||(*y*_1_, *y*_2_)|| for (*y*_1_, *y*_2_) ∈ *K*⋂∂*Ω*_*ρ*_2__.

So, from Lemma 5, *S* has a fixed point $\left(y_{1}^{0},y_{2}^{0})\in K{⋂}_{}({\bar{\Omega }}_{\rho_{2}}\setminus \Omega_{\rho }\right)$ and a fixed point $\left({\bar{y}}_{1},{\bar{y}}_{2})\in K{⋂}_{}({\bar{\Omega }}_{\rho^{*}}\setminus \Omega_{\rho_{2}}\right)$. Both are positive solutions of BVP (1)-(2) with

(35) $$\begin{array}{c} 0<||\left(y_{1}^{0},y_{2}^{0}\right)||<\rho_{2}<||\left({\bar{y}}_{1},{\bar{y}}_{2}\right)||, \end{array}$$

which complete the proof.

### 3.2. Problem (1)–(3) in Case *λ*_1_  =  *λ*_2_  =  1

In the following, for the sake of convenience, set

(36) $$\begin{array}{c} B_{1}:=\max\left\{2\overset{1}{\underset{0}{\int }}G_{1}\left(1,s\right)a_{1}\left(s\right)ds‍,2\overset{1}{\underset{0}{\int }}G_{2}\left(1,s\right)a_{2}\left(s\right)ds‍\right\},\\ B_{2}:=\min\left\{2\overset{1}{\underset{1/2}{\int }}G_{1}\left(1,s\right)a_{1}\left(s\right)ds‍,2\overset{1}{\underset{1/2}{\int }}G_{2}\left(1,s\right)a_{2}\left(s\right)ds‍\right\}. \end{array}$$

Assume that there exist two positive constants *ρ*_1_ ≠ *ρ*_2_ such that

(*H*_3_) *f*(*y*_1_, *y*_2_), *g*(*y*_1_, *y*_2_) ≤ *B*_1_^−1^ *ρ*_1_, for 0 ≤ ||(*y*_1_, *y*_2_)|| ≤ *ρ*_1_;
(*H*_4_) *f*(*y*_1_, *y*_2_), *g*(*y*_1_, *y*_2_) ≥ *B*_2_^−1^ *ρ*_2_, for $\tilde{\gamma }\rho_{2}\leq ||\left(y_{1},y_{2}\right)||\leq \rho_{2}$.

**Theorem 11**

Suppose that (*H*_3_) and (*H*_4_) are satisfied. Then problem (1)-(2), in the case where *λ*_1_ = *λ*_2_ = 1, has at least one positive solution (*y*_1_^0^, *y*_2_^0^) such that ||(*y*_1_^0^, *y*_2_^0^)|| between *ρ*_1_ and *ρ*_2_.

**Proof**

With loss of generality, we may assume that *ρ*_1_ < *ρ*_2_.

Let *Ω*_*ρ*_1__ : = {(*y*_1_, *y*_2_) ∈ *X* : ||(*y*_1_, *y*_2_)|| < *ρ*_1_}. For (*y*_1_, *y*_2_) ∈ *K*⋂_ _∂*Ω*_*ρ*_1__, one has

(37) ||T1(y1,y2)||=λ1∫01G1(1,s)a1(s)f(y1(s),y2(s))ds‍≤B12B1−1ρ1=ρ12=12||(y1,y2)||.

Like Theorem 9, we get ||*S*(*y*_1_, *y*_2_)|| ≤ ||(*y*_1_, *y*_2_)|| for (*y*_1_, *y*_2_) ∈ *K*⋂∂*Ω*_*ρ*_1__.

Now, set *Ω*_*ρ*_2__ : = {(*y*_1_, *y*_2_) ∈ *X* : ||(*y*_1_, *y*_2_)|| < *ρ*_2_}. Then for (*y*_1_, *y*_2_) ∈ *K*⋂∂*Ω*_*ρ*_2__, one has

(38) y1(t)+y2(t)≥min⁡t∈[(1/2),1][y1(t)+y2(t)]≥γ~||(y1,y2)||=γ~ρ2.

Thus, we get

(39) T1(y1,y2)(1)=λ1∫01G1(1,s)a1(s)f(y1(s),y2(s))ds‍≥λ1∫1/21G1(1,s)a1(s)B2−1ρ2ds‍≥B22B2−1ρ2=ρ22=12||(y1,y2)||.

Like Theorem 9, we get ||*S*(*y*_1_, *y*_2_)|| ≥ ||(*y*_1_, *y*_2_)|| for (*y*_1_, *y*_2_) ∈ *K*⋂∂*Ω*_*ρ*_2__. Hence, from Lemma 5, we complete the proof.

**Remark 12**

In [21], problem (1)-(2) with *λ*_1_ = *λ*_2_ = 1 is not considered.

### 3.3. Problem (1), (3) in the General Case

Consider the following.

**Lemma 13 (see [21])**

A pair of functions (*y*_1_, *y*_2_) ∈ *X* is a solution of (1), (3) if and only if (*y*_1_, *y*_2_) is a fixed point of the operator *U* : *X* → *X* defined by

(40) U(y1,y2)(t) :=(U1(y1,y2)(t),U2(y1,y2)(t)) =(β1(t)ϕ1(y1)+λ1∫01G1(t,s)a1(s)‍f×(y1(s),y2(s))ds,β2(t)ϕ2(y2)+λ2∫01G2(t,s)a2(s)g‍×(y1(s),y2(s))ds),

where *β*_1_, *β*_2_ : [0,1]→[0,1] are defined by

(41) $$\begin{array}{c} \beta_{1}\left(t\right):=\frac{\Gamma \left(\nu_{1}-\alpha \right)}{\Gamma \left(\nu_{1}\right)}t^{\nu_{1}-1},\\ \beta_{2}\left(t\right):=\frac{\Gamma \left(\nu_{2}-\alpha \right)}{\Gamma \left(\nu_{2}\right)}t^{\nu_{2}-1}. \end{array}$$

**Lemma 14 (see [21])**

Each of *β*_1_(*t*) and *β*_2_(*t*) is strictly increasing in t and satisfies *β*_1_(0) = *β*_2_(0) = 0 and *β*_1_(1), *β*_2_(1)∈(0,1). Moreover, there exist constants *M*_*β*_1__ and *M*_*β*_2__ satisfying *M*_*β*_1__, *M*_*β*_2__ ∈ (0,1) such that min⁡_*t*∈[(1/2),1]_ *β*_1_(*t*) ≥ *M*_*β*_1__||*β*_1_|| and min⁡_*t*∈[(1/2),1]_ *β*_2_(*t*) ≥ *M*_*β*_2__||*β*_2_||.

Let one define a new cone *K*_1_ by

(42) K1≔{(y1,y2)∈X:y1,y2≥0,min⁡t∈[(1/2),1][y1(t)+y2(t)]≥γ0||(y1,y2)||},

where $\gamma_{0}:=\min\{\tilde{\gamma },M_{\beta_{1}},M_{\beta_{2}}\}$. It is obvious that *γ*_0_ ∈ (0,1).

**Lemma 15 (see [21])**

*U* : *K*_1_ → *K*_1_ is a completely continuous operator.

Now, one assumes

(*D*_1_) *ϕ*_1_(*y*_1_) ≤ ||*y*_1_||/4, *ϕ*_2_(*y*_2_) ≤ ||*y*_2_||/4 for each (*y*_1_, *y*_2_) ∈ *K*_1_;
(*P*_3_) There are numbers Λ_5_,  Λ_6_, where
  (43) Λ5≔max⁡⁡{12[∫1/21γ0G1(1,s)a1(s)ds‍]−1,12[∫1/21γ0G2(1,s)a2(s)ds‍]−1},Λ6≔min⁡⁡{A14[∫01G1(1,s)a1(s)ds‍]−1,A14[∫01G2(1,s)a2(s)ds‍]−1},
 such that Λ_5_ < *λ*_1_,  *λ*_2_ < Λ_6_.
(*P*_4_) There are numbers Λ_7_,  Λ_8_, where
  (44) Λ7≔max⁡⁡{A22[∫1/21G1(1,s)a1(s)ds‍]−1,A22[∫1/21G2(1,s)a2(s)ds‍]−1},Λ8≔min⁡⁡{14[∫01G1(1,s)a1(s)ds‍]−1,14[∫01G2(1,s)a2(s)ds‍]−1},
 such that Λ_7_ < *λ*_1_,  *λ*_2_ < Λ_8_.

**Theorem 16**

Suppose that *f*_0_ = *f*_*∞*_ = *g*_0_ = *g*_*∞*_ = *∞* and (*H*_1_), (*D*_1_), (*P*_3_) are satisfied. Then problem (1), (3) has at least two positive solutions (*y*_1_^0^, *y*_2_^0^),$\;\;({\bar{y}}_{1},{\bar{y}}_{2})$, such that

(45) $$\begin{array}{c} 0<||\left(y_{1}^{0},y_{2}^{0}\right)||<\rho_{1}<||\left({\bar{y}}_{1},{\bar{y}}_{2}\right)||. \end{array}$$

**Proof**

At first, in view of *f*_0_ = *g*_0_ = *∞*, we have *f*(*y*_1_, *y*_2_) ≥ *M*(*y*_1_ + *y*_2_), *g*(*y*_1_, *y*_2_) ≥ *M*(*y*_1_ + *y*_2_), for 0 < ||(*y*_1_, *y*_2_)|| ≤ *ρ*_0_ < *ρ*_1_, where *M* satisfies *M* ≥ 1.

Let *Ω*_*ρ*_0__ : = {(*y*_1_, *y*_2_) ∈ *X* : ||(*y*_1_, *y*_2_)|| < *ρ*_0_}. Then for each (*y*_1_, *y*_2_) ∈ *K*_1_⋂∂*Ω*_*ρ*_0__, we find that

(46) U1(y1,y2)(1)≥λ1∫1/21G1(1,s)a1(s)f(y1(s),y2(s))ds‍≥λ1∫1/21G1(1,s)a1(s)M(y1(s)+y2(s))ds‍≥λ1∫1/21γ0G1(1,s)a1(s)M||(y1,y2)||ds‍≥M2||(y1,y2)||≥12||(y1,y2)||.

So ||*U*_1_(*y*_1_, *y*_2_)|| ≥ (1/2)||(*y*_1_, *y*_2_)|| for (*y*_1_, *y*_2_) ∈ *K*_1_⋂∂*Ω*_*ρ*_0__.

Similarly, we find that ||*U*_2_(*y*_1_, *y*_2_)|| ≥ (1/2)||(*y*_1_, *y*_2_)|| for (*y*_1_, *y*_2_) ∈ *K*_1_⋂∂*Ω*_*ρ*_0__. Consequently,

(47) ||U(y1,y2)||=||(U1(y1,y2),U2(y1,y2))||=||U1(y1,y2)||+||U2(y1,y2)||≥||(y1,y2)||,

whenever (*y*_1_, *y*_2_) ∈ *K*_1_⋂∂*Ω*_*ρ*_0__. Thus, *U* is cone expansion on *K*_1_⋂∂*Ω*_*ρ*_0__.

Next, since *f*_*∞*_ = *g*_*∞*_ = *∞*, we get *f*(*y*_1_, *y*_2_) ≥ *M*_1_(*y*_1_ + *y*_2_), *g*(*y*_1_, *y*_2_) ≥ *M*_1_(*y*_1_ + *y*_2_), for *y*_1_ + *y*_2_ ≥ *ρ*_10_ > *ρ*_1_, where *M*_1_ satisfies *M*_1_ ≥ 1. Let *ρ*_0_* = max⁡{2*ρ*_1_, (*ρ*_10_/*γ*_0_)} and *Ω*_*ρ*_0_*_ : = {(*y*_1_, *y*_2_) ∈ *X* : ||(*y*_1_, *y*_2_)|| < *ρ*_0_*}; then, (*y*_1_, *y*_2_) ∈ *K*⋂∂*Ω*_*ρ*_0_*_ implies

(48) y1(t)+y2(t)≥min⁡t∈[(1/2),1][y1(t)+y2(t)]≥γ0||(y1,y2)||=γ0ρ0∗≥ρ10.

So for (*y*_1_, *y*_2_) ∈ *K*_1_⋂∂*Ω*_*ρ*_0_*_, we obtain

(49) U1(y1,y2)(1)≥λ1∫1/21G1(1,s)a1(s)f(y1(s),y2(s))ds‍≥λ1∫1/21G1(1,s)a1(s)M1(y1(s)+y2(s))ds‍≥λ1∫1/21γ0G1(1,s)a1(s)M1||(y1,y2)||ds‍≥M12||(y1,y2)||≥12||(y1,y2)||.

That is, ||*U*_1_(*y*_1_, *y*_2_)|| ≥ (1/2)||(*y*_1_, *y*_2_)|| for (*y*_1_, *y*_2_) ∈ *K*_1_⋂∂*Ω*_*ρ*_0_*_.

Similarly, we find that ||*U*_2_(*y*_1_, *y*_2_)|| ≥ (1/2)||(*y*_1_, *y*_2_)|| for (*y*_1_, *y*_2_) ∈ *K*_1_⋂∂*Ω*_*ρ*_0_*_. Consequently, ||*U*(*y*_1_, *y*_2_)|| ≥ ||(*y*_1_, *y*_2_)||, whenever (*y*_1_, *y*_2_) ∈ *K*_1_⋂∂*Ω*_*ρ*_0_*_. Thus, *U* is cone expansion on *K*_1_⋂∂*Ω*_*ρ*_0_*_.

Finally, let *Ω*_*ρ*_1__ : = {(*y*_1_, *y*_2_) ∈ *X* : ||(*y*_1_, *y*_2_)|| < *ρ*_1_}. For (*y*_1_, *y*_2_) ∈ *K*⋂∂*Ω*_*ρ*_1__, from (*H*_1_), (*D*_1_), and (*P*_3_), we have

(50) ||U1(y1,y2)||≤ϕ1(y1)+λ1∫01G1(t,s)a1(s)‍×f(y1(s),y2(s))ds≤||y1||4+A14A1−1ρ1≤ρ12=12||(y1,y2)||.

Similarly, we find that ||*U*_2_(*y*_1_, *y*_2_)|| ≤ (1/2)||(*y*_1_, *y*_2_)|| for (*y*_1_, *y*_2_) ∈ *K*_1_⋂∂*Ω*_*ρ*_1__. Consequently, ||*U*(*y*_1_, *y*_2_)|| ≤ ||(*y*_1_, *y*_2_)||, whenever (*y*_1_, *y*_2_) ∈ *K*_1_⋂∂*Ω*_*ρ*_1__. Thus, *U* is cone compression on *K*_1_⋂∂*Ω*_*ρ*_1__.

So, from Lemma 5, *U* has a fixed point $\left(y_{1}^{0},y_{2}^{0})\in K_{1}{⋂}_{}({\bar{\Omega }}_{\rho_{1}}\setminus \Omega_{\rho_{0}}\right)$ and a fixed point $\left({\bar{y}}_{1},{\bar{y}}_{2})\in K_{1}{⋂}_{\;}({\bar{\Omega }}_{\rho_{0}^{*}}\setminus \Omega_{\rho_{1}}\right)$. Both are positive solutions of BVP (1), (3) with

(51) $$\begin{array}{c} 0<||\left(y_{1}^{0},y_{2}^{0}\right)||<\rho_{1}<||\left({\bar{y}}_{1},{\bar{y}}_{2}\right)||. \end{array}$$

The proof is complete.

**Theorem 17**

Suppose that *f*_0_ = *f*_*∞*_ = *g*_0_ = *g*_*∞*_ = 0 and (*H*_2_), (*D*_1_), (*P*_4_) are satisfied. Then problem (1), (3) has at least two positive solutions (*y*_1_^0^, *y*_2_^0^), $\left({\bar{y}}_{1},{\bar{y}}_{2}\right)$, such that

(52) $$\begin{array}{c} 0<||\left(y_{1}^{0},y_{2}^{0}\right)||<\rho_{2}<||\left({\bar{y}}_{1},{\bar{y}}_{2}\right)||. \end{array}$$

**Proof**

At first, in view of *f*_0_ = *g*_0_ = 0, we have *f*(*y*_1_, *y*_2_) < *ε*(*y*_1_ + *y*_2_), *g*(*y*_1_, *y*_2_) < *ε*(*y*_1_ + *y*_2_) for ||(*y*_1_, *y*_2_)|| ≤ *ρ* < *ρ*_2_, where *ε* satisfies *ε* ≤ 1. Let *Ω*_*ρ*_ : = {(*y*_1_, *y*_2_) ∈ *X* : ||(*y*_1_, *y*_2_)|| < *ρ*}. Then for each (*y*_1_, *y*_2_) ∈ *K*_1_⋂_ _∂*Ω*_*ρ*_, we find that

(53) ||U1(y1,y2)|| ≤ϕ1(y1)+λ1∫01G1(t,s)a1(s)f(y1(s),y2(s))ds‍ ≤||y1||4+ε4||(y1,y2)||≤12||(y1,y2)||.

Like Theorem 16, we get ||*U*(*y*_1_, *y*_2_)|| ≤ ||(*y*_1_, *y*_2_)|| for (*y*_1_, *y*_2_) ∈ *K*_1_⋂_ _∂*Ω*_*ρ*_.

Next, in view of *f*_*∞*_ = *g*_*∞*_ = 0, we have *f*(*y*_1_, *y*_2_) < *ε*_1_(*y*_1_ + *y*_2_), *g*(*y*_1_, *y*_2_) < *ε*_1_(*y*_1_ + *y*_2_), for *y*_1_ + *y*_2_ ≥ *ρ*′ > *ρ*_2_, where *ε*_1_ satisfies *ε*_1_ ≤ 1. We consider two cases.

*Case 1.* Suppose that *f* is unbounded and there exists ρ* > *ρ*′ such that

(54) f(y1,y2)≤f(y1∗,y2∗) for  0≤||(y1,y2)||≤ρ∗,||(y1∗,y2∗)||=ρ∗.

Since ρ* > *ρ*′, one has *f*(*y*_1_, *y*_2_) ≤ *f*(*y*_1_*, *y*_2_*) < *ε*_1_(*y*_1_* + *y*_2_*) for 0 ≤ ||(*y*_1_, *y*_2_)|| ≤ ρ*.

Then, for (*y*_1_, *y*_2_) ∈ *K*_1_ and ||(*y*_1_, *y*_2_)|| = ρ*, we obtain

(55) ||U1(y1,y2)|| ≤ϕ1(y1)+λ1∫01G1(t,s)a1(s)f(y1(s),y2(s))ds‍ ≤||y1||4+ε14||(y1,y2)||≤12||(y1,y2)||.

*Case 2.* Suppose that *f* is bounded; there, exists *L*_1_ such that *f*(*y*_1_, *y*_2_) ≤ *L*_1_ for all (*y*_1_, *y*_2_) ∈ *K*_1_. Taking ρ* ≥ max⁡{2*ρ*_2_, *L*_1_}, for (*y*_1_, *y*_2_) ∈ *K*_1_ and ||(*y*_1_, *y*_2_)|| = ρ*, we obtain

(56) ||U1(y1,y2)|| ≤ϕ1(y1)+λ1∫01G1(t,s)a1(s)f(y1(s),y2(s))ds‍ ≤ϕ1(y1)+λ1∫01G1(t,s)a1(s)L1ds‍ ≤||y1||4+L14≤ρ∗2=12||(y1,y2)||.

Hence, in either case, we always may set *Ω*_ρ*_ : = {(*y*_1_, *y*_2_) ∈ *X* : ||(*y*_1_, *y*_2_)|| < ρ*} such that ||*U*_1_(*y*_1_, *y*_2_)|| ≤ (1/2)||(*y*_1_, *y*_2_)|| for (*y*_1_, *y*_2_) ∈ *K*_1_⋂_ _∂*Ω*_ρ*_.

Like Theorem 16, we get ||*U*(*y*_1_, *y*_2_)|| ≤ ||(*y*_1_, *y*_2_)||, for (*y*_1_, *y*_2_) ∈ *K*_1_⋂_ _∂*Ω*_ρ*_.

Finally, set *Ω*_*ρ*_2__ : = {(*y*_1_, *y*_2_) ∈ *X* : ||(*y*_1_, *y*_2_)|| < *ρ*_2_}. Then (*y*_1_, *y*_2_) ∈ *K*_1_⋂_ _∂*Ω*_*ρ*_2__ implies

(57) y1(t)+y2(t)≥min⁡t∈[(1/2),1][y1(t)+y2(t)]≥γ0||(y1,y2)||=γ0ρ2.

Hence we have

(58) U1(y1,y2)(1)≥λ1∫1/21G1(1,s)a1(s)A2−1ρ2ds‍≥A22A2−1ρ2=ρ22=12||(y1,y2)||.

So, ||*U*_1_(*y*_1_, *y*_2_)|| ≥ (1/2)||(*y*_1_, *y*_2_)|| for (*y*_1_, *y*_2_) ∈ *K*_1_⋂_ _∂*Ω*_*ρ*_2__.

Like Theorem 16, we get ||*U*(*y*_1_, *y*_2_)|| ≥ ||(*y*_1_, *y*_2_)|| for (*y*_1_, *y*_2_) ∈ *K*_1_⋂_ _∂*Ω*_*ρ*_2__. So, from Lemma 5, *U* has a fixed point $\left(y_{1}^{0},y_{2}^{0})\in K_{1}{⋂}_{\;}({\bar{\Omega }}_{\rho_{2}}\setminus \Omega_{\rho }\right)$ and a fixed point $\left({\bar{y}}_{1},{\bar{y}}_{2})\in K_{1}{⋂}_{\;}({\bar{\Omega }}_{\rho^{*}}\setminus \Omega_{\rho_{2}}\right)$. Both are positive solutions of BVP (1), (3) with $0<||\left(y_{1}^{0},y_{2}^{0}\right)||<\rho_{2}<||\left({\bar{y}}_{1},{\bar{y}}_{2}\right)||$, which complete the proof.

### 3.4. Problem (1), (3) in Case *λ*_1_  =  *λ*_2_  =  1

In [21], the author obtained that problem (1), (3) with *λ*_1_ = *λ*_2_ = 1 having at least one positive solution. In the following, we also establish the existence of one positive solution to problem (1), (3) with *λ*_1_ = *λ*_2_ = 1 under different conditions.

For the sake of convenience, set

(59) $$\begin{array}{c} B_{3}:=\max\left\{4\overset{1}{\underset{0}{\int }}G_{1}\left(1,s\right)a_{1}\left(s\right)ds‍,4\overset{1}{\underset{0}{\int }}G_{2}\left(1,s\right)a_{2}\left(s\right)ds‍\right\}. \end{array}$$

Assume that there exist two positive constants *ρ*_1_ ≠ *ρ*_2_ such that

(*H*_5_) *f*(*y*_1_, *y*_2_), *g*(*y*_1_, *y*_2_) ≤ *B*_3_^−1^ *ρ*_1_ for 0 ≤ ||(*y*_1_, *y*_2_)|| ≤ *ρ*_1_;
(*H*_6_) *f*(*y*_1_, *y*_2_), *g*(*y*_1_, *y*_2_) ≥ *B*_2_^−1^ *ρ*_2_ for *γ*_0_ *ρ*_2_ ≤ ||(*y*_1_, *y*_2_)|| ≤ *ρ*_2_.

**Theorem 18**

Suppose that (*H*_5_),  (*H*_6_), and (*D*_1_) are satisfied. Then problem (1), (3), in the case where *λ*_1_ = *λ*_2_ = 1, has at least one positive solution (*y*_1_^0^, *y*_2_^0^) such that ||(*y*_1_, *y*_2_)|| between *ρ*_1_ and *ρ*_2_.

**Proof**

With loss of generality, we may assume that *ρ*_1_ < *ρ*_2_. Let *Ω*_*ρ*_1__ : = {(*y*_1_, *y*_2_) ∈ *X* : ||(*y*_1_, *y*_2_)|| < *ρ*_1_}. For (*y*_1_, *y*_2_) ∈ *K*_1_⋂_ _∂*Ω*_*ρ*_1__, from (*H*_7_), (*D*_1_), one has

(60) ||U1(y1,y2)||≤ϕ1(y1)+∫01G1(t,s)a1(s)‍×f(y1(s),y2(s))ds≤ρ14+B34B3−1ρ1=ρ12=12||(y1,y2)||.

Like Theorem 16, we get ||*U*(*y*_1_, *y*_2_)|| ≤ ||(*y*_1_, *y*_2_)|| for (*y*_1_, *y*_2_) ∈ *K*_1_⋂_ _∂*Ω*_*ρ*_1__.

Now, set *Ω*_*ρ*_2__ : = {(*y*_1_, *y*_2_) ∈ *X* : ||(*y*_1_, *y*_2_)|| < *ρ*_2_}. For (*y*_1_, *y*_2_) ∈ *K*_1_⋂_ _∂*Ω*_*ρ*_2__, one has

(61) y1(t)+y2(t)≥min⁡t∈[(1/2),1][y1(t)+y2(t)]≥γ0||(y1,y2)||=γ0ρ2.

Thus, from (*H*_8_), we get

(62) U1(y1,y2)(1)≥λ1∫1/21G1(1,s)a1(s)B2−1ρ2ds‍≥B22B2−1ρ2=ρ22=12||(y1,y2)||.

Like Theorem 16, we get ||*U*(*y*_1_, *y*_2_)|| ≥ ||(*y*_1_, *y*_2_)|| for (*y*_1_, *y*_2_) ∈ *K*_1_⋂_ _∂*Ω*_*ρ*_2__. Hence, from Lemma 5, we complete the proof.

## 4. An Example

To illustrate how our main results can be used in practice, we present one example.

**Example 19**

Consider the following BVP, for *t* ∈ (0,1):

(63) −D0+5.2y1(t)=164500e−2t[(y1(t)+y2(t))1/2+(y1(t)+y2(t))2],−D0+5.95y2(t)=164000e−3t[(y1(t)+y2(t))1/3+(y1(t)+y2(t))3],

subject to the boundary conditions

(64) $$\begin{array}{c} y_{1}^{\left(i\right)}\left(0\right)=y_{2}^{\left(i\right)}\left(0\right)=0,\;0\leq i\leq 4, \end{array}$$

(65) $$\begin{array}{c} D_{0+}^{1.5}{\left[y_{1}\left(t\right)\right]}_{t=1}=D_{0+}^{1.5}{\left[y_{2}\left(t\right)\right]}_{t=1}=0. \end{array}$$

Obviously, problem (63)–(65) fits the framework of problem (1)-(2) with

(66) $$\begin{array}{c} \nu_{1}:=5.2,\;\;\nu_{2}:=5.95,\;\;\alpha =1.5,\\ \lambda_{1}=164500,\;\;\lambda_{2}=164000,\;\;n=6. \end{array}$$

In addition, we have set

(67) $$\begin{array}{c} f\left(y_{1},y_{2}\right)≔{\left(y_{1}+y_{2}\right)}^{1/2}+{\left(y_{1}+y_{2}\right)}^{2},\\ g\left(y_{1},y_{2}\right)≔{\left(y_{1}+y_{2}\right)}^{1/3}+{\left(y_{1}+y_{2}\right)}^{3},\\ a_{1}\left(t\right):=e^{-2t},\;\;a_{2}\left(t\right):=e^{-3t}. \end{array}$$

We can see that *f*, *g* : [0, +*∞*)×[0, +*∞*)→[0, +*∞*) and are continuous. The functions *a*_1_(*t*) and *a*_2_(*t*) are obviously nonnegative.

Now, observe that *f*_0_ = *f*_*∞*_ = *g*_0_ = *g*_*∞*_ = *∞* holds. Again set *A*_1_ = 1/850, because *f*(*y*_1_, *y*_2_), *g*(*y*_1_, *y*_2_) is monotone increasing function for (*y*_1_, *y*_2_) ≥ 0, taking *ρ*_1_ = 4; then, when ||(*y*_1_, *y*_2_)|| ∈ [0, *ρ*_1_], we get

(68) f(y1,y2)≤||(y1,y2)||1/2+||(y1,y2)||2≤2+16=18<4×850=A1−1ρ1g(y1,y2)≤||(y1,y2)||1/3+||(y1,y2)||3≤41/3+64<4×850=A1−1ρ1,

which implies that (*H*_1_) holds.

On the other hand, to calculate the admissible range of the eigenvalues *λ*_1_, *λ*_2_, as given by condition (*P*_1_), observe by numerical approximation, we find that

(69) $$\begin{array}{c} \Lambda_{1}\approx 163530,\;\;\Lambda_{2}\approx 164547.25. \end{array}$$

Thus, for any *λ*_1_, *λ*_2_ satisfying 163530 < *λ*_1_, *λ*_2_ < 164547.25, condition (*P*_1_) will be satisfied.

Consequently, by Theorem 9, problem (63)–(65) has at least two positive solutions.
